# Preservation of Vaccine Virus

**Published:** 1857-09

**Authors:** 


*Preservation of Vaccine Virus.*—A Chicago correspondent of the Peninsular Journal of Medicine, says: "Dr. Johnson read a paper on the use of Glycerine for preserving animal organic cells. He stated that Dr. Andrews, of this city, had made experiments in the preservation of vaccine virus by solution in glycerine, using the solution instead of the solid matter for vaccination, and that by this means the difficult problem of how to preserve the virus in an active state was admirably solved. Dr. Johnson has repeated the experiments of Dr. Andrews with success. He had also examined the solution with the microscope and found the cells perfectly preserved."

"In Dr. Andrews' experiments, the vaccine matter was kept in solution two or three months of warm weather, at the end of which time seven cases were vaccinated with it without a single failure. If this preparation shall prove, as it now seems to promise, to be a permanent form of the virus, it will be of great advantage. The scab broken into three or four pieces is thrown into a little glycerine and occasionally shaken. It will slowly dissolve without any farther care, and almost any number of persons may be vaccinated from the solution, every new scab the practitioner gets may be dropped into the same vial, and thus he will never find himself destitute, as is now the case, by the loss of the active powers of his crusts."

